# Hormonal modulation of reproduction and fertility signaling in polistine wasps

**DOI:** 10.1093/cz/zoab026

**Published:** 2021-03-15

**Authors:** Cintia Akemi Oi, Rafael Carvalho da Silva, Ian Stevens, Helena Mendes Ferreira, Fabio Santos Nascimento, Tom Wenseleers

**Affiliations:** 1 Laboratory of Socioecology and Social Evolution, KU Leuven, Leuven 3000, Belgium; 2 Faculdade de Filosofia, Ciências e Letras de Ribeirão Preto, Departamento de Biologia, Universidade de São Paulo—USP, Ribeirão Preto, SP 14040-901, Brazil

**Keywords:** Polistinae wasps, fertility cues, queen pheromones, cuticular hydrocarbons, juvenile hormone

## Abstract

In social insects, it has been suggested that reproduction and the production of particular fertility-linked cuticular hydrocarbons (CHC) may be under shared juvenile hormone (JH) control, and this could have been key in predisposing such cues to later evolve into full-fledged queen pheromone signals. However, to date, only few studies have experimentally tested this “hormonal pleiotropy” hypothesis. Here, we formally test this hypothesis using data from four species of Polistine wasps, *Polistes dominula*, *Polistes satan*, *Mischocyttarus metathoracicus*, and *Mischocyttarus cassununga*, and experimental treatments with JH using the JH analogue methoprene and the anti-JH precocene. In line with reproduction being under JH control, our results show that across these four species, precocene significantly decreased ovary development when compared with both the acetone solvent-only control and the methoprene treatment. Consistent with the hormonal pleiotropy hypothesis, these effects on reproduction were further matched by subtle shifts in the CHC profiles, with univariate analyses showing that in *P. dominula* and *P. satan* the abundance of particular linear alkanes and mono-methylated alkanes were affected by ovary development and our hormonal treatments. The results indicate that in primitively eusocial wasps, and particularly in *Polistes*, reproduction and the production of some CHC cues are under joint JH control. We suggest that pleiotropic links between reproduction and the production of such hydrocarbon cues have been key enablers for the origin of true fertility and queen signals in more derived, advanced eusocial insects.

In several lineages of highly eusocial insects, specific cuticular hydrocarbons (CHCs) characteristic for queens have been shown to act as worker-sterility inducing queen pheromones, and therefore play a key role in the regulation of their reproductive division of labor (Nunes et al. 2014; Van Oystaeyen et al. 2014; Oi et al. 2015; Holman 2018). Multiple studies indicated that some of these compounds are conserved across different taxa—even in several independent origins of eusociality (Van Oystaeyen et al. 2014; Holman 2018). At present, it is still unclear how these queen signals originated and how they evolved out of cues or signals present in ancestral solitary species (reviewed in Oi et al. 2015). Primitively eusocial species, which are considered a representation of the transition group between ancestral solitary species and advanced eusocial species, provide an interesting opportunity to test hypothesis on how queen pheromones may have first evolved. One hypothesis for why hydrocarbon queen signals are so conserved across different species and lineages is that ancestrally reproduction and the production of particular fertility-linked CHCs was under joint juvenile hormone (JH) control, and that this could have predisposed fertility cues to evolve into full-fledged queen pheromones as well as help to maintain queen signal honesty (Oi et al. 2015; Oliveira et al. 2015, 2017). 

Indeed, in social wasps there is evidence that JH acts both as a gonadotropin by increasing ovary activation and that it controls the production of fertility-linked CHCs (Oi et al. 2015; Oliveira et al. 2017; Table 1). JH is overall known for being tightly connected to reproduction in females because in many insects it triggers the production of vitellogenin (yolk protein precursors) and yolk protein absorption by oocytes (Roy et al. 2018), and thereby acts as a gonadotropin (e.g., in flies, Flatt et al. 2005; Halictid bees, Kapheim and Johnson 2017; crickets, Koch and Hoffmann 1985; and beetles, Trumbo 1997). JH, however, is also known to affect several other functions in insects, JH can regulate metamorphosis or adult behavior (e.g., circadian clock, diapause, sexual behavior, and caste differentiation) (Strambi 1990; Hartfelder and Engels 1998; Nijhout 1998; Hartfelder 2000; Huang 2020; Pandey et al. 2020; Southon et al. 2020). The role of JH in the regulation of the age-related division of labor has also been documented in several other groups of social Hymenoptera, including in the Western honeybee *Apis mellifera* (Hartfelder 2000) and the leaf-cutting ant *Acromyrmex octospinosus* (Norman and Hughes 2016). This makes it a prime example of a hormone with multiple, pleiotropic effects (Flatt et al. 2005; Jindra et al. 2013; Huang 2020). In the Vespine common wasp *Vespula vulgaris* as well as the Epiponini warrior wasp *Synoeca surinama*, such hormonal pleiotropy has also been demonstrated by experiments that showed that JH regulated both fertility and the production of particular fertility-linked hydrocarbons (Kelstrup et al. 2014b; Oliveira et al. 2017; Table 1). The findings that JH affects fertility and the production of fertility cues, however, are certainly not universal in social insects (O’Donnell and Jeanne 1993; Hartfelder 2000; Norman and Hughes 2016). There are many species, for example, where JH lost its original gonadotropic function and instead acquired secondary functions, for example, to help regulate the age-related division of labor or other functions (Robinson 1992; O’Donnell and Jeanne 1993; Hartfelder 2000; Norman and Hughes 2016; Pamminger et al. 2016).

**Table 1.** Review of published literature examining the effects of JH on reproduction (gonadotropic effects), behavior, and the expression of CHC fertility cues in Polistinae and one species of Vespinae wasps

Tribe	*Species*	Gonadotropic effect in (evidence)	Behavioral effect (evidence)	Fertility cues (evidence)	Reference
Vespini	*Vespula vulgaris*	Queens and workers (CE, EM)	/	C_27_, C_28_, C_29_, 3-MeC_29_ (EM—queen pheromone bioassays, CE)	Van Oystaeyen et al. (2014), Oliveira et al. (2017), Oi et al. (2016) and Oi et al. (2020)
Epiponini	*Synoeca surinama*	Dominant females (EM)	Trigger aggression (EM)	C_25:1_, 9-MeC_25_ (EM)	Kelstrup et al. (2014b)
Epiponini	*Polybia occidentalis*	Workers (EM)	Age polyethism (EM)	/	O’Donnell and Jeanne (1993)
Epiponini	*Polybia micans*	Queens (CE)	/	3-MeC_25_; C_25_ (CE)	Kelstrup et al. (2014a)
Polistini	*Polistes annularis*	Workers (EM)	Worker aggression (EM)	/	Barth et al. (1975)
Polistini	*Polistes dominula*	Queenless workers (CE)	Dominance (CE)	/	Tibbetts and Huang (2010)
Polistini	*Polistes dominula*	Foundresses (EM)	/	/	Tibbetts and Izzo (2009)
Polistini	*Polistes dominula*	/	Age polyethism (EM)	/	Shorter and Tibbetts (2009)
Polistini	*Polistes dominula*	/	/	C_31_; C_33_; 13-,15-,17-MeC_33_; 7-MeC_33_; 14-,16-MeC_34_, 13-,17-MeC_35_ (CE); -C29:1, 9-C31:1, n-C33:2, n-C35:2 (CE)	Bonavita-Cougourdan et al. (1991) and Sledge et al. (2001)
Polistini	*Polistes dominula*	Alpha foundresses (CE)	Dominance behavior (CE)	C_29:1_ and C_31:1_ (CE)	Kelstrup et al. (2015)
Polistini	*Polistes smithii*	No effect	Dominance behavior (CE)	None	Kelstrup et al. (2015)
Polistini	*Polistes metricus*	Gynes (EM), foundresses (EM)	/	/	Bohm (1972); Tibbetts and Sheehan (2012)
Polistini	*Polistes canadensis*	Queens (EM)	Worker age polyethism (EM)	/	Giray et al. (2005)
Polistini	*Polistes fuscatus*	Gynes (EM)	Sexual receptivity in gynes (EM)	/	Walton et al. (2020)
Polistini	*Polistes lanio*	/	Sexual maturation in males (EM)	/	Southon et al. (2020)
Mischocyttarini	*Mischocyttarus cerberus*	/	/	5-MeC_29_; C_33:1_, dimeC_31_, MeC_32_, 3-MeC_31_ (CE)	da Silva et al. (2020)
Mischocyttarini	*Mischocyttarus consimilis*	/	/	C_21_, 3-MeC_25_, 13-MeC_29_, 9,21-diMeC33 e 11,15-diMe33 (EM)	Montagna et al. (2015) and Neves et al. (2020)
Ropalidini	*Belonogaster longitarsus*	Gynes (CE)	/	Methyl-branched alkanes with high molecular weight (CE)	Kelstrup et al. (2017)
Ropalidini	*Ropalidia marginata*	(+) Eggs laid by newly emerged females (EM)	Not age polyethism (EM)	/	Agrahari and Gadagkar (2003)

The observed effects of JH have been inferred either from correlative evidence (CE) or experimental manipulation (EM).

In other primitively eusocial wasps, where queens and workers are not strongly morphologically differentiated, there is scattered and sometimes conflicting evidence on the role of JH in influencing reproduction, dominance, behavior, and the expression of chemical fertility cues (Table 1). The dominance hierarchy and ovary activation are often correlated in *Polistes* (Pardi 1948). Quite a few studies in *Polistes* wasps have provided either experimental or correlational evidence for JH acting as a gonadotropic hormone, influencing the reproduction of foundresses, workers, or gynes (Table 1). In *Polistes* paper wasps there is also good correlational evidence for JH influencing dominance behavior in foundresses, which is typically highly correlated with reproduction, as well as experimental evidence for an involvement in regulating worker aggression, sexual receptivity, or worker age polyethism (Table 1). Finally, there is correlational evidence for fertility or ovary activation being linked with the production of particular hydrocarbons in some wasp’ species from Vespini, Polistini, and Epiponini tribes (Table 1), which is common also in other groups of social Hymenoptera and even some solitary insects (Holman et al. 2013; Van Oystaeyen et al. 2014; Oi et al. 2015; Holman 2018). Whether these hydrocarbon cues are actually used as fertility or dominance signals in primitively social wasps is still being investigated or debated (Sledge et al. 2001, 2004; Dapporto et al. 2007, 2010a, 2010b; Izzo et al. 2010; Oi et al. 2019; da Silva et al. 2020), even though fertility-linked CHCs have been shown to act as queen signals in the common wasp *V. vulgaris* (Oliveira et al. 2017). While several studies showed the correlational evidence of chemical differentiation in caste of Polistine wasps, fewer studies conducted experimental manipulation by measuring physiological, behavioral, and chemical expression characteristics simultaneously (Table 1). In particular, the gonadotropic effect of JH was experimentally investigated only for some species of Polistine and studies that check the presence of fertility cues is shown in Table 1.

This study aimed to test consistently the hormonal pleiotropy hypothesis across primitively eusocial wasps, if reproduction and fertility-linked CHCs were under hormonal control in four species of Polistine wasps, *Polistes dominula*, *Polistes satan*, *Mischocyttarus metathoracicus*, and *Mischocyttarus cassununga*. To do that, we compare the species and experimentally treated newly emerged females with JH using the JH analog methoprene and the anti-JH precocene I. Then, we assessed the ovary activation levels and CHC profiles. Looking at primitively eusocial species may shed a light on the evolution of the chemical communication and evolution of fertility signaling.

## Material and Methods

### Experimental setup and study species

The joint effect of JH on fertility (ovary activation) and CHC profiles was investigated in four primitively eusocial Polistine paper wasps: *P. dominula*, *P. satan*, *M. metathoracicus*, and *M. cassununga*. The methodology was adapted as required per species.

#### Polistes dominula

Nineteen post-emergence nests were collected in the vicinity of Leuven (Belgium, 50°53′ N, 4°42′ E) between the end of June and the end of August 2018. Subsequently, all adult individuals were removed, and the nests were glued into wooden boxes (20 × 20 × 13 cm), of which one side was made of Perspex. The nests were kept inside the laboratory under a controlled temperature of 28°C and a 12:12 day/night cycle. Every other day the box was checked for newly emerged individuals. Any newly emerged individuals were treated once with either 2 µL of a methoprene solution (20 µg/µL in acetone, Sigma–Aldrich), 2 µL of a precocene solution (20 µg/µL in acetone, Sigma–Aldrich) or acetone solvent (control group, VWR chemicals), applied topically onto the abdomen. The doses of methoprene and precocene chosen were based on comparable experiments carried out in related species (Izzo et al. 2010; Oliveira et al. 2017). After treatment, the individuals were paint marked according to their treatment using acrylic paint and placed in the nest where they were originated from. Hence, each nest contained individuals of the three groups: treated once with either methoprene, precocene, or acetone sham-treated control. The maximum number of individuals allowed in one box was 20 adults. Whenever this limit was reached, a new box without comb was set up for the remaining individuals. The wasps were fed using mealworms (*Tenebrio molitor*) and sugar water ad libitum. Ten to 12 days after their initial treatment, the individuals were frozen at −20°C to be dissected, in order to assess their ovary activation and to determine their CHC profiles.

#### Polistes satan

Sixteen nests in post-emergence from *P. satan* were collected in Pedregulho (Brazil, 20°15′S, 47°27′W) at the end of February 2018. Adult individuals were removed, and the nests were kept in plastic nest box (18 × 26 × 14 cm). Newly emerged adults were treated once with either 2 µL of a methoprene solution (20 µg/µL in acetone, Sigma–Aldrich), 2 µL of a precocene solution (20 µg/µL, Sigma–Aldrich) or acetone solvent (control group, VWR Chemicals), applied topically onto the abdomen. After treat and paint marking individuals, they were placed back in plastic nest boxes. The nests were kept at similar conditions as *P. dominula* and after 10–12 days the individuals were frozen at −20°C to assess their ovary activation via dissection and determine their CHC profiles.

#### *Mischocyttarus metathoracicus* and *Mischocyttarus cassununga*

A total of 25 nests of *M. metathoracicus* and 8 nests of *M. cassununga* in post-emergence were collected in Ribeirão Preto (Brazil, 21°10′S 47°48′W) in January 2018. Adult individuals were removed and the nest was added into a round plastic container with a diameter of 10 cm. Because of the smaller size of these two species compared with *Polistes* sp. and based on preliminary toxicity assays (results not shown), newly emerged females were treated once with a lower concentration of either methoprene (2 µL of 5 µg/µL in acetone, Sigma–Aldrich), precocene (2 µL of 5 µg/µL in acetone, Sigma–Aldrich) or acetone (2 µL, VWR Chemicals), applied topically onto the abdomen. After 10 days, the individuals were frozen at −20°C to assess their ovary activation via dissection and determine their CHC profiles.

### Ovary activation

**Figure 1.**
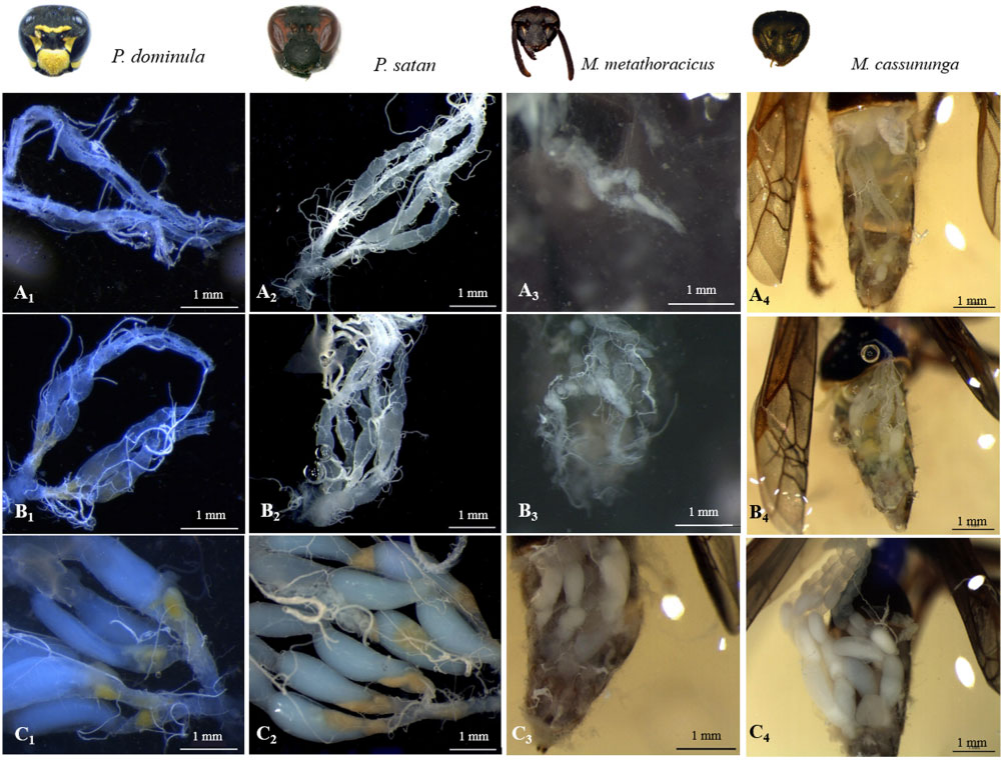
Representative pictures of the three categories of ovary activation found per species. (**A**) Not activated. (**B**) Half activated. (**C**) Fully activated. 1, *Polistes dominula*; 2, *Polistes satan*; 3, *Mischocyttarus metathoracicus*; and 4, *Mischocyttarus cassununga*.

All treated females were dissected under a Leica MZ125 stereomicroscope to assess the ovary activation level. Ovaries were removed from the body and the activation level was visually scored on a three-level ordinal scale, ranging from not activated to half activated or fully activated (Figure 1). Ovaries were considered “not activated” when no activation was observed, meaning that the ovaries were filamentous. The “half activated” ovaries ranged from ovaries that showed little activation, recognized by the presence of thickening oocytes and trophocytes, to ovaries that contained almost fully mature oocytes. The “fully activated” ovaries were those where at least one fully mature, clearly delineated oocyte with a milky white color was present. The difference in ovary activation between treatments was analyzed for all species together using a cumulative link mixed model (CLMM) using the package “ordinal.” The model included treatment and species with interaction as fixed main effects while “nest box” was included as random factor. The model fitted included the data from all four species, in which treatment and species acted additively (on the cumulative logit link scale), that is, with the effect of treatment being identical across species. The model was most parsimonious based on the Akaike Information Criterion value than a model in which the treatment effect was allowed to be different for the different species. ANOVA was performed using the package RVAideMemoire. Tukey’s post hoc comparisons were performed using *lsmeans* (“emmeans” package) to determine significant effects of treatment on ovary scores. No corrections for multiple testing were applied here since the comparisons and predictions were planned a priori. Extra models were conducted for the species separately, including some other covariates that could influence our results (Supplementary Table S1). All statistical analyses were carried in R version 4.0.2.

### Chemical analysis

CHC profiles were determined via GC/MS analysis. Chemical extraction of the cuticular wax layer was performed by immersing whole-body individuals in 3 mL for *Polistes* and 1 mL for *Mischocyttarus* of pentane (ACROS organics) in, respectively, 4 and 1.5 mL GC vials (Agilent) for 1 min. Subsequently, the individuals were removed from the glass vials and the vials were evaporated to near-dryness at room temperature. Subsequently, 150 µL of hexane was added to the vial and the samples were analyzed using coupled gas chromatography/mass spectrometry (Thermo Fisher Trace 1300 ISQ). In the gas chromatograph, 1 µL of sample was injected using splitless injection and separated onto a Restek MXT-5 column (30 m, 0.25 mm of diameter, and 0.25 µm thickness) with helium as carrier gas (constant flow of 0.9 mL min^−1^). The injection temperature was 320°C. Initially, the temperature was held at 40°C for 2 min, then increased to 120°C with a rate of 20°C/min. This was followed by an increase in rate of 10°C/min until 200°C, then 7°C/min to reach 250°C and a last increase of 5°C/min to reach 350°C, which was held for 4 min. Electron impact ionization at 70 eV was used during mass spectrometry at a temperature of 300°C. Linear alkane ladders (Supelco) from C7 to C40 were used as a reference to be able to calculate cubic spline interpolated retention indexes (Messadi et al. 1990). Hydrocarbon peaks were identified on the basis of expected mass spectrometric fragmentation patterns (Carlson et al. 1998) and retention indices by the NIST 2014 retention index database (available online in the NIST Chemistry Webbook—Linstrom and Mallard 2016 or Pherobase El-Sayed 2016). Total ion chromatogram peaks were integrated using R version 4.0.2 using a custom script (available from the authors on request).

To test for the presence of hormonally regulated fertility cues, we compared the log2-transformed relative peak areas of each of the cuticular compounds across the different treatment groups and classes of individuals with a particular level of ovary activation using two-way ANOVAs and LSD post hoc tests with FDR correction (using the aov and lsmeans functions in the “base” and “emmeans” packages of R version 4.0.2). A heatmap (prepared using R’s “pheatmap” package) of the different hydrocarbon compounds was made to compare the mean relative abundance of every compound between the different treatments, using row *z-*scores calculated from the per-treatment class average log2 transformed relative peak areas. Clustering was based on a UPGMA hierarchical clustering using Euclidean distance as the distance metric. The cuticular compounds across the different treatment groups were compared per species using multivariate analyses to highlight possible variations between the groups (treatments and ovary activation). For that, we did a distance matrix using the function *vegdist* and we assessed the similarities of the groups by ANOSIM (distance measure: Bray–Curtis, permutations equal to 999) and PERMANOVA with the *adonis* function in vegan package.

## Results

### Gonadotropic effect of JH

**Figure 2.**
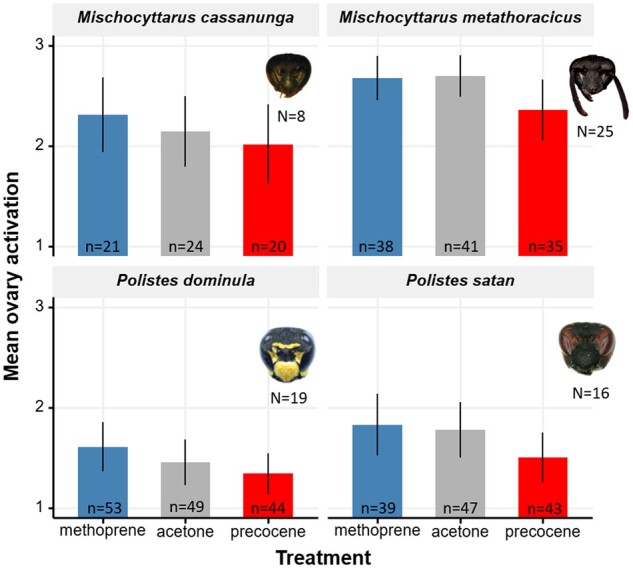
JH has a gonadotropic effect across four primitively eusocial paper wasps. Mean worker ovary activation observed 10–12 days following treatment with the JH analog methoprene and the anti-JH precocene compared with in the acetone solvent control condition in the four paper wasp study species (*Polistes dominula*, *Polistes satan*, *Mischocyttarus metathoracicus*, and *Mischocyttarus cassununga*). Ovaries were scored on a three-level ordinal scale, ranging from not activated (1) to half activated (2) or fully activated (3). Fitted values of a main effects CLMM with nest coded as a random factor with 95% confidence intervals, *N* = number of nests, *n* = total number of individuals treated. Across the four species, precocene significantly decreased ovary development when compared with both the acetone solvent-only control (*z*-ratio = 2.35, *P = *0.02*) and the methoprene treatment (*z*-ratio = 2.96, **P = 0.003). The pairwise between acetone and methoprene was not different (*z*-ratio = −0.75, *P = *0.45) (Table 2C).

Based on a CLMM with species and treatment included as main effects and nest box coded as a random factor (variance = 0.55, standard deviation = 0.73), methoprene did not have a significative effect on ovary activation (*z*-value = 0.65, *P = *0.51) and neither precocene (*z*-value = −0.54, *P = *0.59) (Figure 2 and Table 2A). Species was not significative (Table 2B). Specifically, post hoc tests show that overall, across the four species, precocene have a significant effect decreasing ovary development when compared with both the acetone solvent-only control (*z*-ratio = 2.35, *P = *0.02*) and the methoprene treatment (*z*-ratio = 2.96, *P = *0.003**). The pairwise between acetone and methoprene was not different (*z*-ratio = −0.75, *P = *0.45) (Table 2C).

**Table 2.** (A) Levels of ovary activation across treatments in Polistine wasps were compared using a CLMM with species and treatment included as main effects and nest box coded as a random factor; the model coded arbitrary *M. cassununga* as the reference level in this analysis; (B) ANOVA; and (C) Tukey’s post hoc comparisons were performed to determine significant effects of treatment on ovary scores

(A) Random effects				
Groups names	Variance	*SE*		
Nest (intercept)	0.55	0.73		
Number of groups	68			
Coefficients				
	Estimate	*SE*	*z*-value	Pr(>|*z*|)
Treatment methoprene	0.36	0.56	0.65	0.51
Treatment precocene	−0.30	0.54	−0.54	0.59
Species *Mischocyttarus metathoracicus*	1.61	0.64	2.52	0.01
Species *Polistes dominula*	−1.73	0.61	−2.84	0.00
Species *Polistes satan*	−0.81	0.58	−1.41	0.16
Treatmentmethoprene: species *Mischocyttarus metathoracicus*	−0.49	0.81	−0.60	0.55
Treatmentprecocene: species *Mischocyttarus metathoracicus*	−0.76	0.78	−0.98	0.33
Treatmentmethoprene: species *Polistes dominula*	0.05	0.70	0.07	0.94
Treatmentprecocene: species *Polistes dominula*	−0.12	0.72	−0.17	0.86
Treatmentmethoprene: species *Polistes satan*	−0.33	0.71	−0.47	0.64
Treatmentprecocene: species *Polistes satan*	−0.45	0.70	−0.64	0.52
Threshold coefficients				
	Estimate	*SE*	*z*-value	
Not activated|half activated	−0.34	0.16	−2.12	
Half activated|activated	0.68	0.17	3.98	

(B) Term	LR Chisq	*df*	*P-*value
Treatment	0.00	2	1
Species	0.00	3	1
Treatment: species	1.34	6	0.96

(C) Contrast	estimate	*SE*	*df*	*z*-ratio	*P*-value
Acetone—methoprene	−0.07	0.09	Inf	−0.75	0.45
Acetone—precocene	0.22	0.09	Inf	2.35	0.02^*^
Methoprene—precocene	0.29	0.10	Inf	2.96	0.003^**^

Coefficients, standard errors (SE), *z* values, *P*-values, and degrees of freedom (df) of the random effect intercepts are shown. Significance codes:

*** *P < *0.001,

** *P < *0.01,

* *P < *0.05.

### Hormonally regulated fertility cues

A total of 69 compounds were identified on the cuticle of *P. dominula* (Supplementary Table S2). The chain length of the hydrocarbons ranged from 21 to 37 carbon atoms. This comprised mainly monomethyl, dimethyl, and linear alkanes as well as one trimethyl alkane. In *P. satan*, 41 CHCs were identified, with chain lengths ranging from 23 to 35 carbon atoms (Supplementary Table S3). Here, likewise, compounds comprised mainly monomethyl, dimethyl, and linear alkanes, as well as one trimethyl alkane and two alkenes. In *M. metathoracicus*, a total of 41 compounds were identified (Supplementary Table S4), with chain lengths ranging from 22 to 35 carbon atoms. Here, mainly monomethyl alkanes and linear alkanes occurred with five alkenes and dimethyl alkanes present as well as one trimethyl alkane. Finally, in *M. cassununga* 47 compounds were identified (Supplementary Table S5), with chain lengths ranging from 21 to 33 carbon atoms, composed by linear alkanes, methyl branched alkanes, and dimethyl alkanes present as well as one trimethyl alkane. The difference of chemical compounds between treatment and ovary activation levels was more apparent in *Polistes* than *Michocyttarus* wasps from the multivariate analyses (*P. dominula—*Anosim treatment *R* = 0.15, *P = *0.001; ovary score *R* = 0.05, *P = *0.178; Permanova treatment *R*2 = 0.13, *P = *0.001; ovary score *R*2 = 0.06, *P = *0.0001; *P. satan—*Anosim treatment *R* = 0.05, *P = *0.001; ovary score *R* = 0.00001, *P = *0.503; Permanova treatment *R*2 = 0.05, *P = *0.004; ovary score *R*2 = 0.03, *P = *0.02; *M. cassununga—*Anosim treatment *R* = 0.009, *P = *0.274; ovary score *R* = 0.06, *P = *0.036; Permanova treatment *R*2 = 0.03, *P = *0.372; ovary score *R*2 = 0.06, *P = *0.051 and *M. metathoracicus—*Anosim treatment *R* = 0.01, *P = *0.167; ovary score *R* = −0.02, *P = *0.618; Permanova treatment *R*2 = 0.03, *P = *0.146; ovary score *R*2 = 0.02, *P = *0.269).

**Figure 3.**
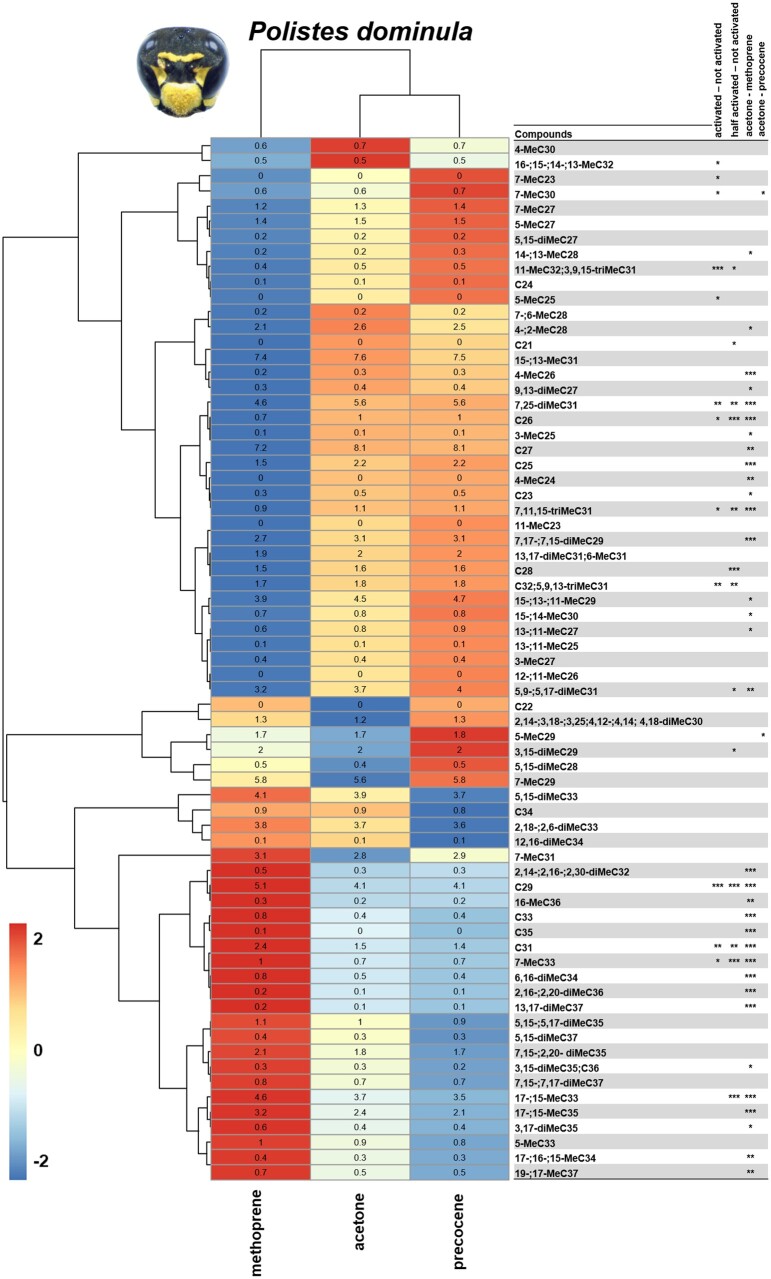
Heatmap showing differences in relative abundance of compounds (log-transformed) in the chemical profiles of *Polistes dominula* females from the three treatments—methoprene, acetone, and precocene. Colors indicate the mean fold difference in relative abundance of each hydrocarbon on the cuticular body surface of treated females. Compounds were clustered based on a UPGMA hierarchical clustering using Euclidean distance as the distance metric. Asterisks indicate the FDR-corrected significances of groups’ contrasts (***P < 0.001, **P < 0.01, *P < 0.05).

The comparison between treatments resulted in subtle shifts in the CHC profiles for *P. dominula*, but not for the other species. The univariate analyses showed that the abundance of particular linear alkanes and mono-methylated alkanes were affected by methoprene treatment. In *P. dominula*, the CHC profiles of methoprene- and acetone-treated individuals differentiate from each other in relative abundance of compounds. The compounds in the lower level of the heatmap are expected to be most linked with an increase of JH since they have a higher relative abundance in the methoprene treatment (Figure 3). Based on the post hoc tests that were performed using the log transformed of the relative area, most of the compounds appear to have a difference in abundance in the methoprene treatment compared with the control, but not for the precocene-treated females. The compounds are most likely to be linked with an increased amount of JH, while they have a low abundance in precocene-treated individuals. The compound differences between acetone and precocene was seen for 7-MeC30, and differences between acetone- and methoprene-treated groups were found for nine linear (C23, C25, C26, C27, C29, C31, C33, C35, and C36), 16 mono-methylated alkanes (4-MeC24, 13-; 11-MeC25, 3-MeC25, 4-MeC26, 3-MeC27, 14-; 13-MeC28, 4-; 2-MeC28, 15-; 13-; 11-MeC29, 15-; 14-MeC30, 7-MeC31, 17-; 15-MeC33, 7-MeC33, 17-; 16-; 15-MeC34, 17-; 15-MeC35, 16-MeC36, and 19-; 17-MeC37), ten (di)methylated alkanes (9,13-diMeC27, 7,17-; 7,15-diMeC29, 7,25-diMeC31, 5,9-; 5,17-diMeC31, 2,14-; 2,16-; 2,30-diMeC32, 6,16-diMeC34, 3,15-diMeC35, 3,17-diMeC35, 2,16-; 2,20-diMeC36, and 13,17-diMeC37), and one (tri)methylated alkane (7,11,15-triMeC31).

**Figure 4.**
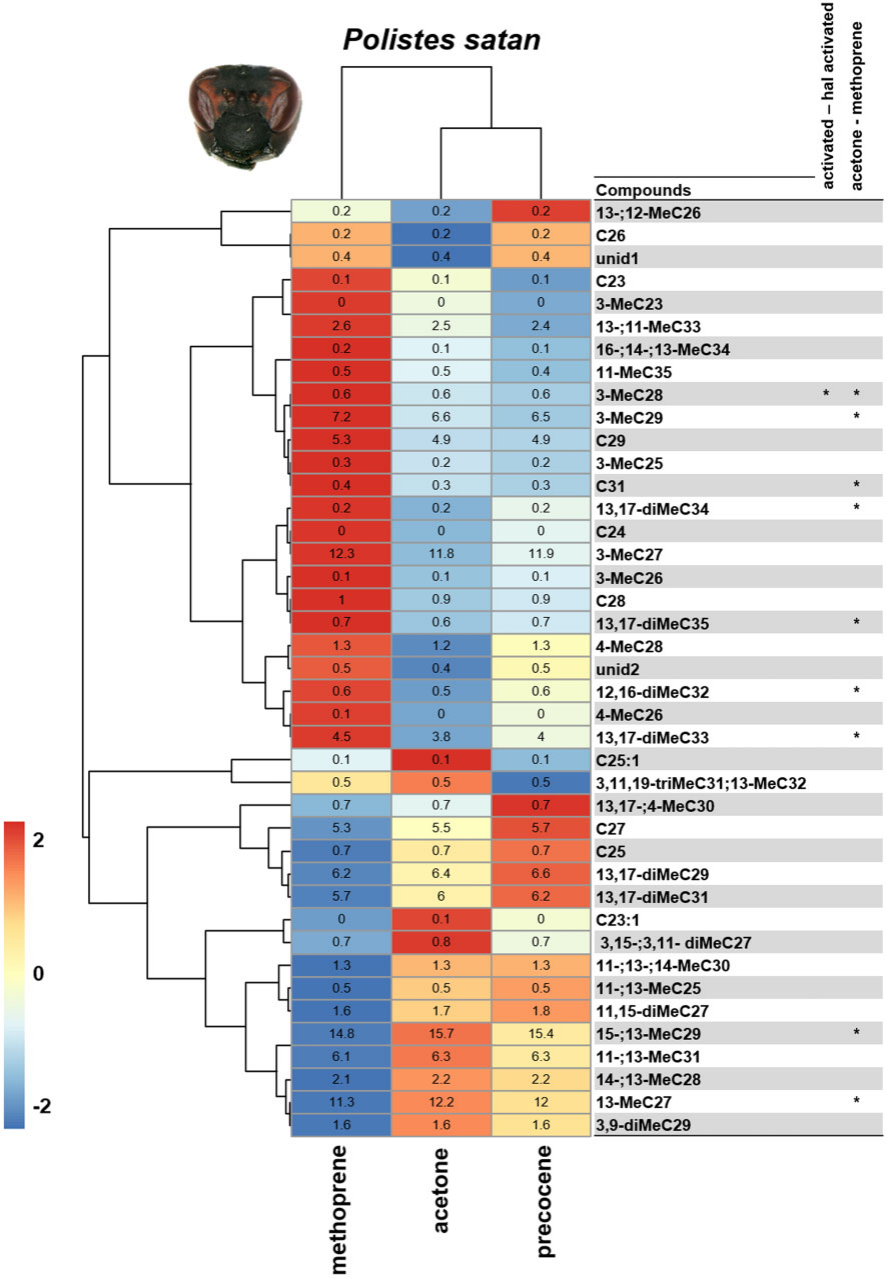
Heatmap showing differences in relative abundance of compounds (log-transformed) in the chemical profiles of *Polistes satan* females from the three treatments—methoprene, acetone, and precocene. Colors indicate the mean fold difference in relative abundance of each hydrocarbon on the cuticular body surface of treated females. Compounds were clustered based on a UPGMA hierarchical clustering using Euclidean distance as the distance metric. Asterisks indicate the FDR-corrected significances of groups’ contrasts (***P < 0.001, **P < 0.01, *P < 0.05).

In *P. satan*, the comparison of CHC profiles between treatments shows that treatment influences the abundance of only a few hydrocarbons. CHCs that are most correlated with the methoprene treatment are shown in the heatmap (Figure 4), significative differences were found for the acetone and methoprene groups for one linear alkane (C31), four mono-methylated alkanes (13-MeC27, 3-MeC28, 15-; 13-MeC29, and 3-MeC29), and four (di)methylated alkanes (12,16-diMeC32, 13,17-diMeC33, 13,17-diMeC34, and 13,17-diMeC35).

**Figure 5.**
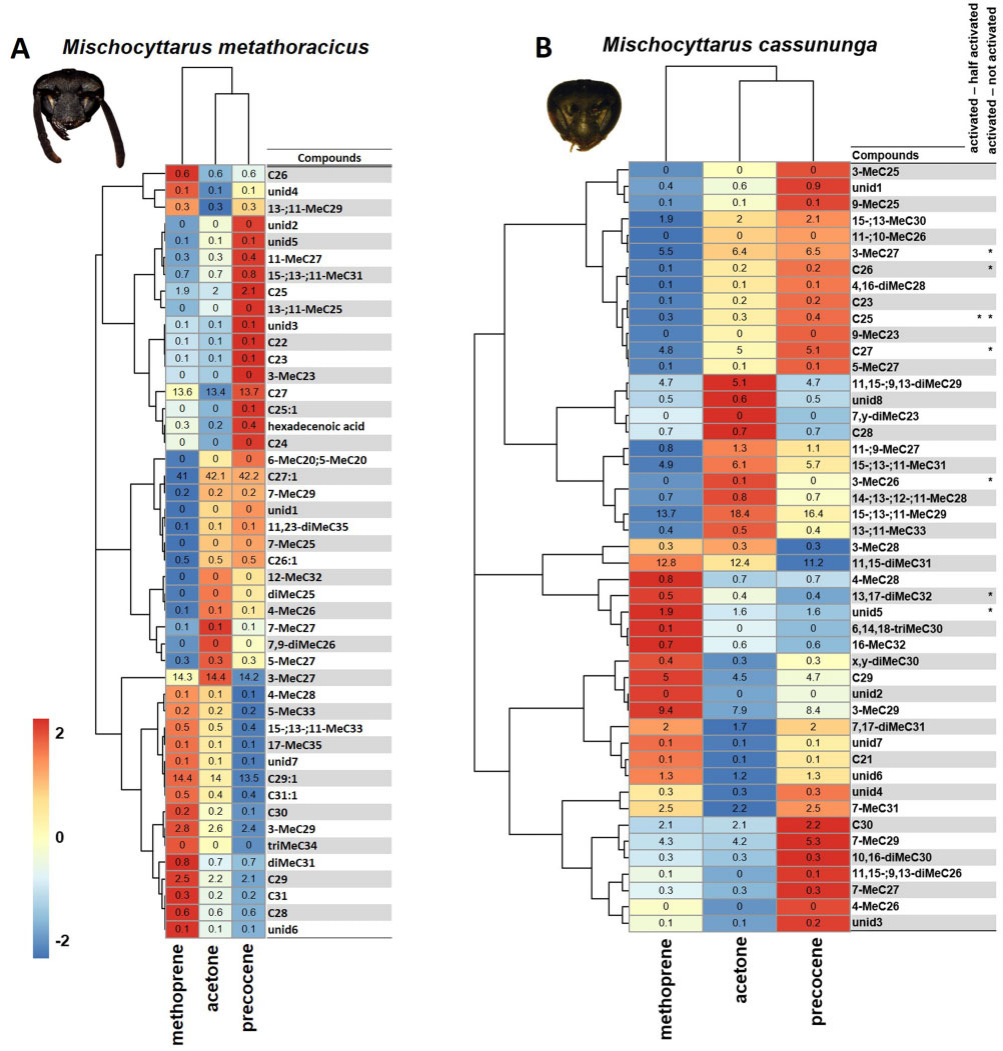
(**A**) Heatmap showing differences in relative abundance of compounds (log-transformed) in the chemical profiles of *Mischocyttarus metathoracicus* females from the three treatments—Methoprene, acetone, and precocene. (**B**) Heatmap showing differences in relative abundance of compounds (log-transformed) in the chemical profiles of *Mischocyttarus cassununga* females. Colors indicate the mean fold difference in relative abundance of each hydrocarbon on the cuticular body surface of treated females. Compounds were clustered based on a UPGMA hierarchical clustering using Euclidean distance as the distance metric. Asterisks indicate the FDR-corrected significances of individual contrasts (***P < 0.001, **P < 0.01, *P < 0.05).

In *M. metathoracicus*, all the individuals across the three treatments (methoprene, acetone, and precocene) appear to have similar CHC profiles. Compounds that are highly correlated with the change in CHC profile in the methoprene treatment are indicated in the heatmap (Figure 5A); however, there was no single compound that differed in the pairwise comparison of methoprene and acetone neither between precocene and acetone. Many of the mean relative hydrocarbon abundances appear to be low in *M. metathoracicus*, while some alkenes, such as C29:1 and C27:1, show a higher abundance. Specifically, the compounds, 3-MeC29, C29, and C29:1 showed an increase of relative abundance in methoprene, that is possibly linked with the amount of JH, even though there is no significant difference in mean abundance between treatments.

Finally, in *M. cassununga*, again, females from the three treatments expressed a high chemical similarity. Compounds that are highly correlated with the change in CHC profile in the methoprene treatment are indicated in the heatmap (Figure 5B), but no single compound was different in pairwise comparison of methoprene and acetone neither precocene nor acetone. Many of the mean relative hydrocarbon abundances appear to be low in *M. cassununga*, while some compounds such as 3-MeC29, C29, and 11,15-diMeC31 show an increase of relative abundance in the methoprene, although not significative, which is possibly linked with the amount of JH.

## Discussion

Our experimental treatments with the JH analogue methoprene and the anti-JH precocene indicate that across our four primitively eusocial paper wasp study species JH indeed acts as a gonadotropin, with the precocene treatment significantly reducing ovary activation levels, there being a trend toward increased ovary activation levels in the methoprene-treated females and there being no evidence for the four study species responding differently to these hormone treatments. This is consistent with earlier correlational and experimental evidence for JH acting as a gonadotropin in social wasps (Table 1). In line with the hormonal pleiotropy hypothesis, our experimental treatments also induced subtle shifts in the CHC profiles, particularly in *Polistes*, with univariate analyses showing that in *P. dominula* and *P. satan*, the abundance of particular linear alkanes and mono-methylated alkanes were affected by our hormonal treatments, particularly by the methoprene application, and that some of these compounds were also markers of ovary activation, that is, fertility cues. In our two *Mischocyttarus* study species, however, we only found evidence for the presence of fertility-linked hydrocarbon cues in one of the two study species, in *M. cassununga*, and no evidence for either these CHC compounds or any others to be influenced by our hormone treatments. Hence, overall, we found clear evidence in *Polistes* for the hormonal pleiotropy hypothesis and for ovary activation and the production of CHC fertility cues to be under joint hormonal control, but not in *Mischocyttarus*.

The specific CHCs that we found to be fertility-linked and whose production we found to be regulated by the JH-analog methoprene were the long-chain linear alkanes C29 and C31 and the methyl-branched alkane 7-MeC33 in *P. dominula* and the 3-methyl-branched alkane 3-MeC28 in *P. satan*. A number of studies previously identified a larger set of fertility-linked compounds in *P. dominula* and *P. satan*, and several of those compounds also overlap with the compounds we find to be overproduced following methoprene-treatment (in *P. dominula*: C33, 7 + 15 + 17-MeC33, which are more abundant on the cuticle of foundresses compared with workers, Bonavita-Cougourdan et al. 1991, and the alkenes C29:1 and C31:1, which were previously more abundant on the cuticle and eggs of reproductively dominant foundresses, Kelstrup et al. 2015; in *P. satan*: C31 and 3-MeC29, which were both more abundant on the cuticle of reproductively dominant foundresses, Tannure-Nascimento et al. 2008; Oi et al. 2019; Table 1). In our two *Mischocyttarus* study species, both C29 and 3-MeC29 also showed a trend to be overproduced following methoprene treatment, though neither of these reached statistical significance, and we also found no evidence for these CHCs to be linked with ovary activation in these cases (in *Mischocyttarus*, typically most females in the nest have active ovaries, making it hard to find a significant association with the production of particular CHC cues, da Silva et al. 2020). Also, in *M. metathoracicus*, the alkene C29:1 was overproduced in the methoprene-treated wasps, but again no significance was reached. Overall, for all the studied species, the occurrence of C29 and 3-MeC29 as JH-regulated fertility cues is interesting from the fact that in the eusocial wasp *V. vulgaris*, the hydrocarbons C29 and 3-MeC29 were also among the bioactive worker sterility-inducing queen pheromones (Van Oystaeyen et al. 2014). This adds credence to the hypothesis that queen pheromones in advanced eusocial species evolved from fertility cues in primitively eusocial ones (Van Oystaeyen et al. 2014; Oi et al. 2015, 2019). In addition, the hormonal pleiotropy that we document, and which causes reproduction and the production of particular fertility-linked CHCs to be under joint JH control, would also be expected to be a key enabler for fertility cues to evolve into full-fledged queen pheromones and should also help to maintain queen signal honesty (Oi et al. 2015; Oliveira et al. 2015, 2017).

Although our results show that JH acts as a gonadotropin in our primitively eusocial wasp study species, we cannot exclude that JH could also exert other effects in other contexts, as posited by the so-called split-function hypothesis (Turillazzi and West-Eberhard 1996). This hypothesis states that the effect of JH could depend on the social context and nutritional state (Turillazzi and West-Eberhard 1996). Indeed, in several primitively eusocial wasps, such context-dependent effects of JH have been well documented. In the Epiponini swarm-founding wasp *Polybia occidentalis*, for example, treatment of female workers with the JH-analog methoprene accelerated the workers’ age polyethism in the presence of queens, while it led to increased reproduction in their absence (O’Donnell and Jeanne 1993). Likewise, in *P. dominula* foundresses, JH had a comparatively bigger effect on dominance and fertility in larger females (Shorter and Tibbetts 2009; Tibbetts and Izzo 2009), while in *P. canadensis*, JH only affected reproduction in gynes, due to their greater nutritional reserves, but not in workers, where it instead resulted in a faster transition of behaviors from nursing to guarding and foraging (Giray et al. 2005). Such context-dependent endocrine effects may be an adaptive strategy that enable females to allocate more energy toward dominance and fertility if they have a greater likelihood to become future reproductives (Tibbetts and Izzo 2009).

One of the caveat of our study is that we did not differentiate the newly emerged females, being gynes or workers, this could have influenced our results due to the nutritional differences of the adults and the susceptibility of the JH treatments. Furthermore, another methodological restriction could be the quantification of methoprene and precocene used to treat the species, it may not be ideal due to the fact that little is known about the circulating levels of JH. Another caveat was comparing primitively eusocial species from temperate and tropical areas, because the environment biological conditions in which species occur affect their colonial cycle. Females of *P. dominula* exhibit a strong overwintering behavior (West-Eberhard 1969), while females of *P. satan* express a mild overwinter aggregation behavior (Tannure-Nascimento et al. 2005). The overwintering behavior seems to be absent in tropical *Mischocyttarus* species (Torres et al. 2011; Biagiotto and Shima 2017). In *Mischocyttarus*, there is a high frequency of females with activated ovaries from pre-worker emergence to post-worker emergence nests (Murakami et al. 2009; da Silva et al. 2020), and relatively small number of females in later stage compared with *Polistes.* The differences between the four studied species could be explained by context dependency—species that still retain a strong overwintering behavior or seasonality, such as *Polistes* are different from asynchronous colonial cycles such as *Mischocyttarus*, although this is rather speculative.

In conclusion, we demonstrated that JH has a conserved gonadotropic effect in four unrelated species of primitively eusocial wasps. In addition, we are able to demonstrate that JH is also responsible for regulating the production of fertility chemical cues. The fact that hormonal manipulations have a significant effect on ovary activation support the hormonal pleiotropy hypothesis, although changes in chemical composition were more pronounced in *Polistes* rather than *Mischocyttarus* species. Future behavioral experiments investigating the effects of JH and interactions between individuals should be considered, especially for *Mischocyttarus* species, for instance performing observation in situ natural conditions, it would be important to check whether behavioral interactions in the colonial context trigger physiological responses (increasing levels of JH or ovary activation), how the circulating levels of JH of different females change depending on the stage of the nest (first or last workers to emerge), how important individual condition (fat body) is and play a role on individual sensibility to JH treatments and how JH influence behavioral changes (from aggressive acts related to their social dominance to age-related task progression).

## Acknowledgments

The authors thank An Vandoren for chemical analyses assistance and students who help them with the experiments.

## Author Contributions

C.A.O. designed the experiments. C.A.O., I.S., R.C.d.S., and H.M.F. performed the experiments and chemical analysis. C.A.O., F.S.N., and T.W. provide the funding. C.A.O., R.C.d.S., I.S., and T.W. wrote the manuscript. All authors contributed to revise and proofreading the manuscript.

## Supplementary Material

zoab026_Supplementary_Data

## References

[zoab026-B1] Agrahari M, Gadagkar R, 2003. Juvenile hormone accelerates ovarian development and does not affect age polyethism in the primitively eusocial wasp *Ropalidia marginata*. J Insect Physiol 49**:** 217–222. 12769996 10.1016/s0022-1910(02)00268-8

[zoab026-B3] Barth RH, Lester LJ, Sroka P, Kessler T, Hearn R, 1975. Juvenile-hormone promotes dominance behavior and ovarian development in social wasps *Polistes annularis*. Experientia 31**:** 691–692. 1170088 10.1007/BF01944632

[zoab026-B5] Biagiotto RH, Shima SN, 2017. Comparative study of the development of *Mischocyttarus cassununga* Von Ihering and *Mischocyttarus cerberus styx* Richards colonies (Hymenoptera, Vespidae, Mischocyttarini). EntomoBrasilis 10**:** 170–177.

[zoab026-B6] Bohm MK, 1972. Effects of environment and juvenile hormone on ovaries of the wasp *Polistes metricus*. J Insect Physiol 18**:** 1875–1883.

[zoab026-B7] Bonavita-Cougourdan A, Theraulaz G, Bagnères AG, Roux M, Pratte M et al, 1991. Cuticular hydrocarbons, social organization and ovarian development in a polistine wasp: *polistes dominulus* Christ. Comp Biochem Physiol B Biochem Mol Biol 100**:** 667–680.

[zoab026-B9] Carlson DA, Bernier UR, Sutton BD, 1998. Elution patterns from capillary GC for methyl-branched alkanes. J Chem Ecol 24**:** 1845–1865.

[zoab026-B10] da Silva RC, Prato A, Oi CA, Turatti ICC, Nascimento FS, 2020. Dominance hierarchy, ovarian activity and cuticular hydrocrabons in the primitively eusocial wasp *Mischocyttarus cerberus* (Vespidae, Polistinae, Mischocyttarini). J Chem Ecol 46**:** 835–844. 32789711 10.1007/s10886-020-01206-1

[zoab026-B11] Dapporto L, Bruschini C, Cervo R, Dani FR, Jackson DE et al, 2010a. Timing matters when assessing dominance and chemical signatures in the paper wasp *Polistes dominulus*. Behav Ecol Sociobiol 64**:** 1363–1365.

[zoab026-B12] Dapporto L, Bruschini C, Cervo R, Petrocelli I, Turillazzi S, 2010b. Hydrocarbon rank signatures correlate with differential oophagy and dominance behaviour in *Polistes dominulus* foundresses. J Exp Biol 213**:** 453. 20086130 10.1242/jeb.032938

[zoab026-B13] Dapporto L, Santini A, Dani FR, Turillazzi S, 2007. Workers of a *Polistes* paper wasp detect the presence of their queen by chemical cues. Chem Senses 32**:** 795. 17644826 10.1093/chemse/bjm047

[zoab026-B15] El-Sayed AM, 2016. *The Pherobase: Database of Insect Pheromones and Semiochemicals* [Internet]. Available from http://www.pherobase.com.

[zoab026-B16] Flatt T, Tu M-P, Tatar M, 2005. Hormonal pleiotropy and the juvenile hormone regulation of *Drosophila* development and life history. Bioessays 27**:** 999–1010. 16163709 10.1002/bies.20290

[zoab026-B21] Giray T, Giovanetti M, West-Eberhard MJ, 2005. Juvenile hormone, reproduction, and worker behavior in the neotropical social wasp *Polistes canadensis*. Proc Natl Acad Sci U S A 102**:** 3330–3335. 15728373 10.1073/pnas.0409560102 PMC552932

[zoab026-B22] Hartfelder K, 2000. Insect juvenile hormone: from “status quo” to high society. Braz J Med Biol Res 33**:** 157–177. 10657056 10.1590/s0100-879x2000000200003

[zoab026-B23] Hartfelder K, Engels W, 1998. Social insect polymorphism: hormonal regulation of plasticity in development and reproduction in the honeybee. Curr Top Dev Biol 40**:** 45–77. 9673848 10.1016/s0070-2153(08)60364-6

[zoab026-B24] Holman L, 2018. Queen pheromones and reproductive division of labor: a meta-analysis. Behav Ecol 29**:** 1199–1209.

[zoab026-B25] Holman L, Lanfear R, d’Ettorre P, 2013. The evolution of queen pheromones in the ant genus *Lasius*. J Evol Biol 26**:** 1549–1558. 23662630 10.1111/jeb.12162

[zoab026-B26] Huang ZY, 2020. Juvenile hormone. In: Starr CK, editor. Encyclopedia of Social Insects. Cham: Springer International Publishing. 1–3.

[zoab026-B28] Izzo A, Wells M, Huang Z, Tibbetts E, 2010. Cuticular hydrocarbons correlate with fertility, not dominance, in a paper wasp, *Polistes dominulus*. Behav Ecol Sociobiol 64**:** 857–864.

[zoab026-B29] Jindra M, Palli SR, Riddiford LM, 2013. The juvenile hormone signaling pathway in insect development. Annu Rev Entomol 58:181–204. 22994547 10.1146/annurev-ento-120811-153700

[zoab026-B30] Kapheim KM, Johnson MM, 2017. Juvenile hormone, but not nutrition or social cues, affects reproductive maturation in solitary alkali bees *Nomia melanderi*. J Exp Biol 220**:** 3794–3801. 28821570 10.1242/jeb.162255

[zoab026-B32] Kelstrup HC, Hartfelder K, Esterhuizen N, Wossler TC, 2017. Juvenile hormone titers, ovarian status and epicuticular hydrocarbons in gynes and workers of the paper wasp *Belonogaster longitarsus*. J Insect Physiol 98**:** 83–92. 27913150 10.1016/j.jinsphys.2016.11.014

[zoab026-B33] Kelstrup HC, Hartfelder K, Nascimento FS, Riddiford LM, 2014a. Reproductive status, endocrine physiology and chemical signaling in the Neotropical, swarm-founding eusocial wasp *Polybia micans*. J Exp Biol 217**:** 2399–2410. 24744417 10.1242/jeb.096750 PMC4081010

[zoab026-B34] Kelstrup HC, Hartfelder K, Nascimento FS, Riddiford LM, 2014b. The role of juvenile hormone in dominance behavior, reproduction and cuticular pheromone signaling in the caste-flexible epiponine wasp *Synoeca surinama*. Front Zool 11**:** 1–19. 25371699 10.1186/s12983-014-0078-5 PMC4219083

[zoab026-B35] Kelstrup HC, Hartfelder K, Wossler TC, 2015. *Polistes smithii* vs. *Polistes dominula*: the contrasting endocrinology and epicuticular signaling of sympatric paper wasps in the field. Behav Ecol Sociobiol 69**:** 2043–2058.

[zoab026-B36] Koch PB, Hoffmann KH, 1985. Juvenile hormone and reproduction in crickets, *Gryllus bimaculatus* De Geer: corpus allatum activity (in vitro) in females during adult life cycle. Physiol Entomol 10**:** 173–182.

[zoab026-B38] Linstrom PJ, Mallard WG, 2016. *NIST Chemistry Webbook* [Internet]. Gaithersburg, MD: National Institute of Standards and Technology. Available from http://webbook.Nist.Gov.

[zoab026-B39] Messadi D, Helaimia F, Ali-Mokhnache S, Boumahraz M, 1990. Accurate determination of retention indices in programmed temperature gas chromatography. Chromatographia 29**:** 429–434.

[zoab026-B41] Montagna TS, Raizer J, Antonialli WF, 2015. Effect of larval topical application of juvenile hormone on caste determination in the independent–founding eusocial wasp *Mischocyttarus consimilis* (Hymenoptera: Vespidae). Open J Anim Sci 5**:** 174–184.

[zoab026-B42] Murakami ASN, Shima SN, Desuó IC, 2009. More than one inseminated female in colonies of the independent-founding wasp *Mischocyttarus cassununga* von Ihering (Hymenoptera. Vespidae). Rev Bras Entomol 53**:** 653–662.

[zoab026-B43] Neves EF, Montagna TS, Junior LCS, Michelutti KB, Cardoso CAL et al, 2020. Effect of larval topical application of juvenile hormone on cuticular chemical composition of *Mischocyttarus consimilis* (Vespidae: Polistinae) females. Sociobiology 67**:** 433–443.

[zoab026-B44] Nijhout HF, 1998. Insect Hormones. Princeton, New Jersey: Princeton University Press.

[zoab026-B45] Norman VC, Hughes WOH, 2016. Behavioural effects of juvenile hormone and their influence on division of labour in leaf-cutting ant societies. J Exp Biol 219**:** 8–11. 26739685 10.1242/jeb.132803

[zoab026-B46] Nunes TM, Mateus S, Favaris AP, Amaral MF, von Zuben LG et al, 2014. Queen signals in a stingless bee: suppression of worker ovary activation and spatial distribution of active compounds. Sci Rep 4**:** 7449. 25502598 10.1038/srep07449 PMC4264003

[zoab026-B47] O’Donnell S, Jeanne RL, 1993. Methoprene accelerates age polyethism in workers of a social wasp *Polybia occidentalis*. Physiol Entomol 18:189–194.

[zoab026-B48] Oi CA, Brown RL, Da Silva RC, Wenseleers T, 2020. Reproduction and signals regulating worker policing under identical hormonal control in social wasps. Sci Rep 10**:** 18971. 33149171 10.1038/s41598-020-76084-4 PMC7643062

[zoab026-B49] Oi CA, Millar JG, van Zweden JS, Wenseleers T, 2016. Conservation of queen pheromones across two species of vespine wasps. J Chem Ecol 42**:** 1175–1180. 27722875 10.1007/s10886-016-0777-9

[zoab026-B50] Oi CA, Oliveira RC, van Zweden JS, Mateus S, Millar JG et al, 2019. Do primitively eusocial wasps use queen pheromones to regulate reproduction? A case study of the paper wasp *Polistes satan*. Front Zool 7**:** 199.

[zoab026-B51] Oi CA, van Zweden JS, Oliveira RC, Van Oystaeyen A, Nascimento FS et al, 2015. The origin and evolution of social insect queen pheromones: novel hypotheses and outstanding problems. Bioessays 37**:** 808–821. 25916998 10.1002/bies.201400180

[zoab026-B52] Oliveira RC, Oi CA, Nascimento MMC, Vollet-Neto A, Alves DA et al, 2015. The origin and evolution of queen and fertility signals in corbiculate bees. BMC Evol Biol 15**:** 254. 26573687 10.1186/s12862-015-0509-8 PMC4647589

[zoab026-B53] Oliveira RC, Vollet-Neto A, Akemi Oi C, van Zweden JS, Nascimento F et al, 2017. Hormonal pleiotropy helps maintain queen signal honesty in a highly eusocial wasp. Sci Rep 7**:** 1654. 28490760 10.1038/s41598-017-01794-1 PMC5431770

[zoab026-B54] Pamminger T, Treanor D, Hughes WOH, 2016. Pleiotropic effects of juvenile hormone in ant queens and the escape from the reproduction-immunocompetence trade-off. Proc R Soc B Biol Sci 283**:** 20152409. 10.1098/rspb.2015.2409 PMC4721097 26763704

[zoab026-B55] Pandey A, Motro U, Bloch G, 2020. Juvenile hormone affects the development and strength of circadian rhythms in young bumble bee *Bombus terrestris* workers. Neurobiol Sleep Circadian Rhythms 9**:** 100056. 33364524 10.1016/j.nbscr.2020.100056 PMC7752729

[zoab026-B56] Pardi L, 1948. Dominance order in *Polistes* wasps. Physiol Zool 21**:** 1–13. 18898533 10.1086/physzool.21.1.30151976

[zoab026-B58] Robinson GE, 1992. Regulation of division of labor in insect societies. Annu Rev Entomol 37**:** 637–665. 1539941 10.1146/annurev.en.37.010192.003225

[zoab026-B62] Roy S, Saha TT, Zou Z, Raikhel AS, 2018. Regulatory pathways controlling female insect reproduction. Annu Rev Entomol 63**:** 489–511. 29058980 10.1146/annurev-ento-020117-043258

[zoab026-B64] Shorter JR, Tibbetts EA, 2009. The effect of juvenile hormone on temporal polyethism in the paper wasp *Polistes dominulus*. Insectes Soc 56**:** 7–13.

[zoab026-B65] Sledge MF, Boscaro F, Turillazzi S, 2001. Cuticular hydrocarbons and reproductive status in the social wasp *Polistes dominulus*. Behav Ecol Sociobiol 49**:** 401–409.

[zoab026-B66] Sledge MF, Trinca I, Massolo A, Boscaro F, Turillazzi S, 2004. Variation in cuticular hydrocarbon signatures, hormonal correlates and establishment of reproductive dominance in a polistine wasp. J Insect Physiol 50**:** 73–83. 15037095 10.1016/j.jinsphys.2003.10.001

[zoab026-B67] Southon RJ, Radford AN, Sumner S, 2020. Hormone-mediated dispersal and sexual maturation in males of the social paper wasp *Polistes lanio*. J Exp Biol 223**:** jeb226472. 33139391 10.1242/jeb.226472

[zoab026-B68] Strambi A, 1990. Physiology and reproduction in social wasps. In: Engels W, editor. Social Insects: An Evolutionary Approach to Castes and Reproduction. Heidelberg: Spring. 59–75.

[zoab026-B69] Tannure-Nascimento I, Nascimento F, Zucchi R, 2005. Size and colony cycle in *Polistes satan*, a Neotropical paper wasp (Hymenoptera Vespidae). Ethol Ecol Evol 17**:** 105–119.

[zoab026-B70] Tannure-Nascimento IC, Nascimento FS, Zucchi R, 2008. The look of royalty: visual and odour signals of reproductive status in a paper wasp. Proc R Soc Lond B Biol Sci 275**:** 2555. 10.1098/rspb.2008.0589 PMC2605800 18682372

[zoab026-B73] Tibbetts EA, Huang ZY, 2010. The challenge hypothesis in an insect: juvenile hormone increases during reproductive conflict following queen loss in *Polistes* wasps. Am Nat 176**:** 123–130. 20515411 10.1086/653664

[zoab026-B74] Tibbetts EA, Izzo AS, 2009. Endocrine mediated phenotypic plasticity: condition-dependent effects of juvenile hormone on dominance and fertility of wasp queens. Horm Behav 56**:** 527–531. 19751736 10.1016/j.yhbeh.2009.09.003

[zoab026-B76] Tibbetts EA, Sheehan MJ, 2012. The effect of juvenile hormone on *Polistes* wasp fertility varies with cooperative behavior. Horm Behav 61**:** 559–564. 22349082 10.1016/j.yhbeh.2012.02.002

[zoab026-B77] Torres VO, Montagna TS, Fernandes WD, Antonialli-Junior WF, 2011. Colony cycle of the social wasp *Mischocyttarus consimilis* Zikán (Hymenoptera, Vespidae). Rev Bras Entomol 55**:** 247–252.

[zoab026-B78] Trumbo ST, 1997. Juvenile hormone-mediated reproduction in burying beetles: from behavior to physiology. Arch Insect Biochem Physiol 35**:** 479–490.

[zoab026-B80] Turillazzi S, West-Eberhard MJ, 1996. Natural History and Evolution of Paper-Wasps. Oxford: Oxford University Press.

[zoab026-B81] Van Oystaeyen A, Oliveira RC, Holman L, Van Zweden JS, Romero C et al, 2014. Conserved class of queen pheromones stops social insect workers from reproducing. Science 287**:** 287–290. 10.1126/science.1244899 24436417

[zoab026-B83] Walton A, Tumulty JP, Toth AL, Sheehan MJ, 2020. Hormonal modulation of reproduction in *Polistes fuscatus* social wasps: dual functions in both ovary development and sexual receptivity. J Insect Physiol 120**:** 103972. 31705844 10.1016/j.jinsphys.2019.103972 PMC7558460

[zoab026-B84] West-Eberhard MJ, 1969. The Social Biology of Polistine Wasps. Ann Harbor: University of Michigan.

