# Supplementary material for: Hormonal modulation of reproduction and fertility signaling in polistine wasps
Source: Curr Zool. 2021 Mar 15;67(5):519–30. doi: 10.1093/cz/zoab026 (PMC8489163; doi:10.1093/cz/zoab026)
Supplement: zoab026_Supplementary_Data [file zoab026_supplementary_data.pdf]

Supplementary Material

Hormonal modulation of reproduction and fertility signalling in Polistine wasps

Cintia Akemi OI[a*], Rafael Carvalho da SILVA[b], Ian STEVENS[a], Helena Mendes FERREIRA[a], Fabio Santos NASCIMENTO[b] and Tom WENSELEERS[a]

[a]Laboratory of Socioecology and Social Evolution, KU Leuven, Leuven, Belgium; [b]Universidade de São Paulo – USP/ Faculdade de Filosofia, Ciências e Letras de Ribeirão Preto, Departamento de Biologia, SP, Ribeirão Preto, Brazil.

*Address correspondence to Cintia Akemi Oi. Email: cintiaakemioi@gmail.com

Handling editor: Alessandro Cini

Received on 18 September 2020; accepted on 10 March 2021

Table S1.Ovary activation between treatments was analysed for the Polistine species separately using a Cumulative Link Mixed Model (CLMM). Individuals models included treatment as fixed main effect while nest was included as random factor. The covariates tested in the different species are included in specifically in each of the models and are indicated for each species. Coefficients, standard errors (SE), z values and *p* values.

(A) *Polistes dominula* model with presence of comb size and head width as covariates

**Random effects**		
Groups name	variance	SE
nest (intercept)	0.75	0.87
Number of groups	19	

**Coefficients**	estimate	SE	z value	Pr(>|z|)
Treatment methoprene	0.00	0.26	0.02	0.98
Treatment precocene	-0.39	0.25	-1.56	0.12
comb	0.06	0.25	0.12	0.91
head_width	-2.15	1.49	-1.45	0.15

**Threshold coefficients**	estimate	SE	z value
not activated|half activated	-9.97	5.30	-1.88
half activated|activated	-8.61	5.28	-1.63

(B) *Polistes dominula* model with total number of larvae as covariate

**Random effects**		
Groups name	Variance	SE
nest (intercept)	2.23	1.49
Number of groups	11	

**Coefficients**	estimate	SE	z value	Pr(>|z|)
Treatment methoprene	-0.97	0.44	-2.16	0.03*
Treatment precocene	-0.54	0.42	-1.29	0.19
total_larvae	0.017	0.26	0.66	0.51

**Threshold coefficients**	Estimate	SE	z value
not activated|half activated	-1.60	1.54	-1.04
half activated|activated	-0.36	1.55	-0.23

(C) *Polistes satan* model with head width as covariate

**Random effects**		
Groups name	variance	SE
nest (intercept)	0.20	0.45
Number of groups	16	

**Coefficients**	estimate	SE	z value	Pr(>|z|)

treatmentmethoprene	-0.23	0.25	-0.93	0.35
treatmentprecocene	-0.23	0.26	-0.89	0.37
head_width	-1.82	1.04	1.76	0.08

**Threshold coefficients**	estimate	SE	z value
not activated|half activated	-9.81	4.96	-1.98
half activated|activated	-9.01	4.95	-1.82

(D) *Mischocyttarus metathoracicus* model with number of pupae as covariate

**Random effects**		
Groups name	Variance	SE
nest (intercept)	0.20	0.45
Number of groups	25	

**Coefficients**	estimate	SE	z value	Pr(>|z|)
Treatment methoprene	0.35	0.33	1.07	0.28
Treatment precocene	0.25	0.32	0.77	0.44
pupae	0.09	0.05	1.70	0.09

**Threshold coefficients**	estimate	SE	z value
not activated|half activated	-0.60	0.51	-1.17
half activated|activated	-0.33	0.50	-0.65

(E) *Mischocyttarus cassanunga* model with number of pupae as covariate

**Random effects**		
Groups name	Variance	Std. Dev
nest (intercept)	0.62	0.79
Number of groups	8	

**Coefficients**	Estimate	Std. Error	z value	Pr(>|z|)
Treatment methoprene	-0.01	0.33	-0.04	0.97
Treatment precocene	0.44	0.36	1.23	0.21
pupae	0.02	0.04	0.54	0.59

**Threshold coefficients**	Estimate	Std. Error	z value
not activated|half activated	-0.92	0.78	-1.18
half activated|activated	1.02	0.80	1.29

Table S2. Identified cuticular hydrocarbons of *Polistes dominula*. Retention time, retention index and diagnostic ions were used to identify the hydrocarbons

	**Compound**	**Retention time**	**Retention index**	**diagnostic ions**
1	C21	18.01	2096	296
2	C22	19.08	2194	310
3	C23	20.16	2293	324
4	11-MeC23	20.56	2329	168, 196
5	7-MeC23	20.62	2335	112, 252
6	C24	21.24	2392	338
7	4-MeC24	21.92	2455	71, 308
8	C25	22.32	2492	352
9	13-;11-MeC25	22.69	2526	168, 169, 224, 225
10	5-MeC25	22.87	2542	112, 280, 309
11	3-MeC25	23.11	2564	337, 57
12	C26	23.42	2591	336
13	12-;11-MeC26	23.79	2624	182,168, 196, 238
14	4-MeC26	24.13	2654	70, 71, 336, 337
15	C27	24.58	2693	380
16	13-;11-MeC27	24.92	2724	196, 224
17	7-MeC27	25.03	2733	112, 309
18	5-MeC27	25.14	2742	85, 337
19	9,13-diMeC27	25.27	2754	295, 183, 112, 252
20	3-MeC27	25.39	2764	56365
21	5,15-diMeC27	25.48	2773	351, 239, 84, 196
22	C28	25.71	2792	394
23	14-;13-MeC28	26.05	2822	210, 196, 224, 238
24	7-;6-MeC28	26.2	2835	112, 323
25	4-;2-MeC28	26.44	2855	71, 365
26	5,15-diMeC28	26.59	2868	365
27	C29	26.89	2894	409
28	15-;13-;11-MeC29	27.23	2924	225, 196, 252
29	7-MeC29	27.36	2936	112, 336
30	5-MeC29	27.47	2944	84364
31	7,17-;7,15-diMeC29	27.69	2964	351, 267; 196, 239, 224
32	3,15-diMeC29	28.04	2994	407, 239, 56, 224
33	15-;14-MeC30	28.37	3023	224, 238, 210, 252
34	7-MeC30	28.45	3030	112, 350
35	4-MeC30	28.75	3057	394, 71
36	2,14-;3,18-;3,25-diMeC30	28.92	3071	435, 235, 42, 224
37	C31	29.2	3096	437
38	15-;13-MeC31	29.53	3124	224, 252
39	7-MeC31	29.66	3136	112, 365
40	13,17-diMeC31;6-meC31	29.77	3145	225, 197, 239, 267, 295, 365
41	7,25-diMeC31	29.97	3164	379, 112

42	5,9-;5,17-diMeC31	30.09	3174	379, 450, 113
43	7,11,15-triMeC31	30.22	3185	393, 183, 112, 323, 252
44	C32; 5,9,13-triMeC31	30.34	3196	464
45	16-;15-;14-;13-MeC32	30.64	3223	224, 210, 238, 252, 266, 295
46	11-MeC32;3,9,15-triMeC31	30.69	3228	252, 169
47	2,14-;2,16-;2,30-diMeC32	31.21	3273	225,280,408,464
48	C33	31.46	3296	465
49	17-;15-MeC33	31.77	3324	224, 252, 280
50	7-MeC33	31.9	3336	112, 422, 394
51	5-MeC33	32.01	3347	85, 422
52	2,18-;2,6-diMeC33	32.2	3364	478, 281, 42, 238
53	5,15-diMeC33	32.32	3375	435, 239, 84, 280
54	C34	32.57	3398	478
55	17-;16-;15-MeC34	32.85	3423	238,252,224,266, 280, 294
56	12,16-diMeC34	33.18	3454	253, 280, 436
57	6,16-diMeC34	33.29	3465	435, 253, 98, 280
58	C35	33.63	3496	492
59	17-,15-MeC35	33.93	3524	252, 280, 224, 308
60	3,17-diMeC35	34.19	3548	196, 267, 351, 280
61	7,15-;2,20- diMeC35	34.35	3564	435, 239, 112, 308
62	5,15-;5,17-diMeC35	34.46	3574	464, 239, 84, 308
63	3,15-diMeC35;C36	34.72	3600	280, 211, 308, 351, 492
64	16-MeC36	34.99	3625	308, 281, 407, 71
65	2,16-;2,20-diMeC36	35.43	3668	308, 464, 235, 281, 255
66	19-,17-MeC37	36.02	3725	280, 252, 308
67	13,17-diMeC37	36.27	3750	196, 267, 308, 379
68	7,15-;7,17-diMeC37	36.43	3765	464, 239, 112, 196, 407, 168
69	5,15-diMeC37	36.54	3775	492, 239, 84, 336

Table S3. Identified cuticular hydrocarbons of *Polistes satan*. Retention time, retention index and diagnostic ions were used to identify the hydrocarbons

	Compound	Retention time	Retention index	diagnostic ions
1	C23:1	21.56	2276	322
2	C23	21.84	2300	324
3	3-MeC23	22.72	2372	57, 58, 309, 308
4	C24	23.04	2399	338
5	C25:1	23.95	2473	350
6	C25	24.25	2497	352
7	11-;13-MeC25	24.67	2531	168, 169, 224, 225
8	3-MeC25	25.17	2571	56,57,336,337
9	C26	25.5	2597	366
10	13-;12-MeC26	25.92	2630	196, 210; 182, 224
11	4-MeC26	26.24	2656	70, 336
12	3-MeC26	26.43	2670	56, 350
13	C27	26.76	2697	380
14	13-MeC27	27.18	2730	196, 197, 224, 225
15	unid1	27.4	2747	/
16	11,15-diMeC27	27.52	2757	168, 186, 239, 267
17	3-MeC27	27.72	2773	56, 57, 364, 365
18	C28	28.02	2797	349
19	3,15-;3,11- diMeC27	28.1	2803	379, 239, 56, 196
20	14-;13-MeC28	28.42	2828	210, 224; 196, 238
21	4-MeC28	28.76	2855	70, 71, 364, 365
22	3-MeC28	28.96	2871	56, 57, 378, 379
23	C29	29.29	2897	408
24	15-;13-MeC29	29.7	2930	224; 196, 252
25	13,17-diMeC29	29.99	2953	421, 196, 266
26	3-MeC29	30.24	2973	407, 56, 392/393
27	3,9-diMeC29	30.61	3003	407, 155, 56, 308
28	11-;13-;14-MeC30	30.93	3029	168,294
29	13,17-diMeC30;4-MeC30	31.25	3055	70, 71, 392, 393
30	C31	31.77	3098	436
31	11-,13-MeC31	32.17	3130	168,308 ; 196,280
32	13,17-diMeC31	32.45	3153	295, 267, 196, 224
33	3,11,19-triMeC31;13-MeC32	33.36	3228	450, 196, 309; 196, 294
34	12,16-diMeC32	33.65	3253	323, 253, 182, 252
35	13-;11-MeC33	34.55	3329	463, 196, 308 ;168, 336
36	13,17-diMeC33	34.83	3353	196, 252, 267, 323
37	unid2	35.11	3377	/
38	16-;14-;13-MeC34	35.73	3430	182, 336, 323, 196, 210
39	13,17- diMeC34	36	3454	337, 267, 196, 266
40	11-MeC35	36.86	3530	168, 169, 364, 365
41	13,17-diMeC35	37.12	3553	351, 267, 196, 280

Table S4. Identified cuticular hydrocarbons of *Mischocyttarus metathoracicus*. Retention time, retention index and diagnostic ions were used to identify the hydrocarbons

	Compound	Retention time	Retention index	diagnostic ions
1	unid1	17.88	1971	/
2	5-;6-MeC20	18.6	2031	224, 225; 210, 211
3	unid2	20.41	2181	/
4	C22	20.59	2197	310
5	C23	21.8	2298	324
6	3-MeC23	22.69	2372	57, 58, 309, 308
7	C24	23	2398	338
8	C25:1	23.93	2474	350
9	C25	24.21	2496	352
10	13-;11-MeC25	24.64	2531	168, 169, 224, 225
11	7-MeC25	24.73	2539	112, 113, 280, 281
12	C26:1	25.18	2574	334
13	C26	25.45	2596	336
14	4-MeC26	26.2	2656	70, 71, 336, 337
15	C27:1	26.5	2679	83, 97, 111, 379
16	C27	26.74	2699	380
17	11-MeC27	27.15	2731	196, 224
18	7-MeC27	27.24	2739	112, 309
19	5-MeC27	27.36	2748	85, 337
20	3-MeC27	27.68	2773	56, 57, 364, 365
21	C28	27.99	2798	394
22	unid3	28.48	2837	/
23	4-MeC28	28.73	2857	70, 71, 364, 365
24	C29:1	29.02	2880	406
25	C29	29.26	2899	409
26	13-;11-MeC29	29.68	2932	225, 196, 252
27	7-MeC29	29.79	2941	112, 336
28	3-MeC29	30.21	2975	407, 56, 392
29	C30	30.53	3001	422
30	C31:1	31.54	3082	435
31	C31	31.77	3101	436
32	15-;13-;11-MeC31	32.15	3132	168,308 ; 196,280
33	diMeC31	32.69	3177	/
34	unid4	33.04	3206	/
35	12-MeC32	33.38	3235	182, 183, 308, 309
36	triMeC34	34.19	3303	/
37	15-;13-;11-MeC33	34.56	3334	463, 196, 308 ;168, 336
38	5-MeC33	34.9	3363	85, 422
39	unid5	35.1	3381	/
40	17-MeC35	36.9	3538	252, 280
41	11,23-diMeC35	37.25	3569	379, 351, 168, 196

Tables S5. Identified cuticular hydrocarbons of *Mischocyttarus cassununga*. Retention time, retention index and diagnostic ions were used to identify the hydrocarbons

	Compound	Retention time	Retention index	diagnostic ions
1	C21	19.39	2096	296
2	unid1	19.99	2146	41, 55, 69
3	C23	21.77	2297	324
4	9-MeC23	22.24	2335	141,225
5	unid2	22.84	2385	41, 55, 69
6	7,y-diMeC23	22.98	2396	267
7	C25	24.18	2495	352
8	9-MeC25	24.63	2531	141,253
9	3-MeC25	25.09	2568	57,337
10	C26	25.42	2594	366
11	11-;10-MeC26	25.84	2628	169,239/155,253
12	4-MeC26	26.15	2652	71,337
13	3-MeC26	26.35	2668	71,351
14	11,15-;9,13-diMeC26	26.40	2672	253, 267, 281
15	C27	26.68	2694	380
16	11-;9-MeC27	27.09	2727	169,253/141,280
17	7-MeC27	27.19	2735	112, 309
18	5-MeC27	27.31	2744	85,337
19	3-MeC27	27.61	2768	57,365
20	C28	27.93	2794	394
21	unid3	28.01	2800	168, 267
22	14-;13-;12-;11-MeC28	28.34	2826	169,267/183,253/197,239/211,225
23	unid4	28.42	2833	69, 81, 112
24	4-MeC28	28.67	2852	71,365
25	3-MeC28	28.86	2868	57,379
26	4,16-diMeC28	29.05	2883	379 253 70 196
27	C29	29.19	2894	408
28	15-;13-;11-MeC29	29.61	2928	169,351, 197,253, 225
29	7-MeC29	29.72	2936	113,337
30	11,15-; 9,13-diMeC29	29.95	2954	295 239 168 224, 323 211 140 252
31	3-MeC29	30.14	2969	57,393
32	C30	30.52	3000	435
33	15-;13-MeC30	30.83	3025	197,267/239,225
34	unid5	31.17	3053	
35	10,16-diMeC30	31.53	3082	224, 253
36	x,y-diMeC30	31.67	3094	211 239 267
37	6,14,18-triMeC30	31.89	3111	436
38	15-;13-;11-MeC31	32.06	3125	169,309/197,281/225,253
39	7-MeC31	32.18	3135	112, 365
40	11,15-diMeC31	32.40	3153	168, 239, 252, 323
41	7,17-diMeC31	32.66	3174	379 267 112 224
42	unid6	32.95	3199	196, 222, 267
43	16-MeC32	33.31	3229	238, 253
44	13,17-diMeC32	33.60	3253	309 267 196 238
45	unid7	33.78	3268	224, 281
46	13-;11-MeC33	34.46	3326	168, 197, 295, 337
47	unid8	34.77	3353	
